# Exosomal CD63 in critically ill patients with sepsis

**DOI:** 10.1038/s41598-021-99777-w

**Published:** 2021-10-13

**Authors:** Yunjoo Im, Hongseok Yoo, Ryoung-Eun Ko, Jin Young Lee, Junseon Park, Kyeongman Jeon

**Affiliations:** 1 Division of Pulmonary and Critical Care Medicine, Department of Medicine, Samsung Medical Center, Sungkyunkwan University School of Medicine, 81 Irwon-ro, Gangnam-gu, Seoul, 06351 Republic of Korea; 2 Department of Critical Care Medicine, Samsung Medical Center, Sungkyunkwan University School of Medicine, Seoul, Republic of Korea

**Keywords:** Predictive markers, Molecular medicine

## Abstract

CD63 is one of the tetraspanin protein family members that is ubiquitously expressed on exosomes and is involved in the signal transduction of various types of immune cells. It may thus contribute to immunometabolic mechanisms of cellular and organ dysfunction in sepsis. Nonetheless, the association of exosomal CD63 with the severity and mortality of sepsis is not well known. Therefore, in the present study, the overall levels of exosomal CD63 were evaluated to ascertain whether they were associated with organ failure and mortality in patients with sepsis. Exosomal CD63 was measured from prospectively enrolled critically-ill patients with sepsis (n = 217) and healthy control (n = 20). To detect and quantify exosomes in plasma, a commercially available enzyme-linked immunosorbent assay kit was used according to the manufacturer’s protocol. The total number of exosomal CD63 was determined by quantifying the immunoreactive CD63. The association between plasma levels of exosomal CD63 and sequential organ failure assessment (SOFA) score was assessed by a linear regression method. The best cut-off level of exosomal CD63 for 28-day mortality prediction was determined by Youden’s index. Among 217 patients with sepsis, 143 (66%) patients were diagnosed with septic shock. Trends of increased exosomal CD63 levels were observed in control, sepsis, and septic-shock groups (6.6 µg/mL *vs*. 42 µg/mL *vs*. 90 µg/mL, p < 0.001). A positive correlation between exosomal CD63 and SOFA scores was observed in patients with sepsis (*r* value = 0.35). When patients were divided into two groups according to the best cut-off level, the group with higher exosomal CD63 levels (more than 126 µg/mL) was significantly associated with 28-day and in-hospital mortality. Moreover, the Kaplan–Meier survival method showed a significant difference in 90-day survival between patients with high- and low-exosomal CD63 levels (log-rank p = 0.005). Elevated levels of exosomal CD63 were associated with the severity of organ failure and predictive of mortality in critically ill patients with sepsis.

## Introduction

Sepsis is a life-threatening inflammatory response syndrome caused by a dysregulated host response to infection with an uncertain pathophysiology^1^. Sepsis can lead to septic shock owing to the circulatory and cellular metabolism abnormalities that are adequate to increase mortality^1^. Given that there is currently no gold standard diagnostic test, there is increasing awareness that biomarkers of sepsis, including transcriptomic, metabolomic, proteomic, are essential to understand the pathophysiology of sepsis^2,3^. New sepsis biomarkers will likely lead to a better characterization of sepsis and may be proven helpful in the determination of organ dysfunction and evaluation of the patients’ clinical courses^4,5^.

In a recent study, exosomes, the smallest type of extracellular vesicles (size range, 30–100 nm), were affiliated with the severity of organ failure and mortality prediction in critically ill patients with sepsis^6^. Growing evidence suggests that components of exosome, such as proteins or ribonucleic acids (RNAs) from different origins are associated with organ failure^7–10^. The tetraspanin protein family members, such as CD63, CD81, and CD9, ubiquitously expressed on exosomes and extensively used as exosome biomarkers, are involved in physiological processes, for instance cell adhesion, cell motility, and signal transduction^11,12^. CD63, the first characterized tetraspanin, has two extracellular loops of unequal sizes and two short cytoplasmic domains, is involved in the signal transduction processes of various types of immune cells, and may contribute to the immunometabolic mechanisms of cellular and organ dysfunction in sepsis^13–15^. A number of studies implicated CD63 in intracellular transport of pathogens, such as bacteria and viruses into the endosomal system^16–20^. In this regard, the utility of CD63 as a biomarker for diagnosis and prognosis of sepsis has been a particular field of interest. However, the role of exosomal CD63 was not confirmed in humans, and the association of exosomal CD63 with the severity of organ failure and mortality in critical ill patients have not evaluated yet. Therefore, we assessed the levels of exosomal CD63 in sepsis patients and evaluated whether these were associated with organ failure and mortality.

## Materials and methods

The data used in this study were derived from an ongoing single center prospective registry of critical illness patients from tertiary referral centers in Seoul, South Korea (Samsung Medical Center, 1989 beds, university affiliated). This cohort was started in April 2014 for the establishment of a human sample repository and development of new biological markers for critical illness^21^. Informed consent including the research purpose, achievement of clinical data, blood specimen, and future reporting of collected data prior to enrollment was obtained from all study participants or their legal representatives. This study was approved by the institutional review board of the Samsung Medical Center and performed in compliance with Helsinki declaration.

### Study population

The protocols of patient enrollment and data collection have been described previously in our earlier research study^6,22,23^. Briefly, patients aged 19 years or older admitted to the medical intensive care unit (ICU) were prospectively enrolled, and baseline demographics, clinical details—including severity of illness scoring collected in the first 24 h after admission to the ICU, laboratory data, and relevant outcomes—were recorded. As this is an ongoing cohort, we included a total of 217 patients with sepsis from April 2014 to January 2019, who admitted to the medical ICU. The diagnosis of sepsis was based on the guidelines of the third International Consensus Definitions for Sepsis and Septic Shock (Sepsis-3)^1^. Given that the enrollment for the registry began in April 2014, we reclassified patients who included before release of the new definition. In addition, 20 healthy controls (older than 19 years) donated their blood specimen (5 mL each) for purposes of investigation. Healthy controls who are at least 19 years old, willing and capable of providing informed consent were included. Participants with a clinically significant abnormal laboratory value and/or clinically significant unstable medical or psychiatric illness were excluded.

### Quantification of exosomal CD63

We collected whole blood from each study participant within 48 h of study enrollment, isolated plasma aliquots from each sample by centrifuging at 480×*g* and 4 °C for 10 min, and stored them at − 80 °C. To separate exosome from plasma, ExoQuick exosome precipitation solution (System Biosciences, Palo Alto, CA, USA) and enzyme-linked immunosorbent assay kit (Novusbio, Littleton, CO, USA) were used. To minimize contamination of isolated exosomes, we pre-treated plasma samples with thrombin to remove debris, and filtered samples with a 0.2 μm filter to remove larger vesicles prior to using the commercial kit, as recommended in a previous study compared the purity and yields of isolated exosomes in several commercial kit^24^. To characterize isolated exosomes, we used transmission electron microscopy to assess the size of morphology of exosomes, and conducted western blot to investigate the presence of tetraspanins^6^. Moreover, flow cytometry analysis was carried out using exosome isolation and analysis kit (Abcam, Cambridge, MA, USA) to demonstrate of the presence of CD63 on the surface of exosome.

The protein concentration of the isolated exosomes was determined using a Pierce BCA protein assay kit (Thermo Scientific, Waltham, MA, USA), according to the manufacturer’s instructions. A standard curve was derived with nine points of serial dilution with bovine serum albumin and a working reagent. All samples and standard points were replicated three times. And then, the total number of exosomal CD63 was determined by quantifying the immunoreactive CD63 (ExoELISA kit, System Biosciences, Mountain View, CA, USA). CD63, lysed exosomal protein were bounded in the plate, then, anti-CD63 antibody and secondary antibody were added respectively to quantify the amount of CD63 presence in exosome.

### Statistical analysis

Baseline characteristics of participants were summarized as numbers and proportions for categorical variables, and median with interquartile range (IQR, 25th–75th percentiles) for continuous variables. Preliminary analysis compared the outcome based on these categorical variables using the Chi-square or Fisher’s exact tests, and continuous variables using the Mann–Whitney *U* test. The exosomal CD63 levels in control, sepsis, and septic-shock groups were compared with the Kruskal–Wallis test.

A linear regression analysis was performed to estimate the associations between exosomal CD63 and severity of organ failure, as measured by the sequential organ failure assessment (SOFA) score^25^. Receiver operating characteristic analysis was conducted to evaluate the predictive ability of exosomal CD63 level as a prognostic predictor of disease severity. The optimal cut-off points for discriminating between the exosomal CD63 level and 28-day mortality in our cohort were calculated with Youden’s index^26^. We reclassified patients based on the optimal cutoff level of exocomal CD63, into two groups of high and low-exosomal CD63. Accordingly, the initial diagnosis, clinical status, severity of illness, and 28-day mortality associated with the two groups were compared*.* The Kaplan–Meier equation was used to determine the 90-day mortality curves according to the exosomal CD63 levels. These levels were then compared with the log-rank test.

All statistical analyses were performed using R version 3.5.3 (R Foundation for Statistical Computing, http://www.r-project.org). Two-sided p-values < 0.05 were considered statistically significant.

## Results

Baseline characteristics of all participants are listed in Table 1. During the study period, 143 (66%) patients were diagnosed septic shock. Eighty eight (41%) patients received mechanical ventilation and 163 (75%) patients were on vasopressor support. To assess the severity of illness, SAPS (p < 0.001), APACHE II (p = 0.028) and SOFA (p < 0.001) scores were quantified and were found higher in patients with septic shock compared with sepsis patients. However, no significant difference was observed in 28-day, in-hospital, and 90-day mortalities between the two groups.

**Table 1:** Characteristics of study participants.

	Total (n = 217)	Sepsis (n = 74)	Septic shock (n = 143)	p*-*value
Age, years	67 (55–74)	66 (52–76)	67 (58–73)	0.931
Sex, male	149 (69)	54 (73)	95 (66)	0.406
BMI, kg/m^2^	22.8 (20.3–25.6)	22.6 (19.7–25.0)	23.0 (20.4–25.7)	0.165
**Comorbidity**				
Diabetes mellitus	71 (33)	20 (27)	51 (36)	0.257
Coronary heart disease	9 (4)	3 (4)	6 (4)	0.999
Chronic kidney disease	16 (7)	6 (8)	10 (7)	0.981
Solid tumor	104 (48)	35 (47)	69 (48)	0.999
Hematologic malignancy	31 (14)	13 (18	18 (13)	0.430
Charlson comorbidity index	2 (1–3)	2 (1–3)	2 (1–3)	0.736
**Clinical status on ICU admission**				
Need for mechanical ventilation	88 (41)	25 (34)	63 (44)	0.188
Need for vasopressor support	163 (75)	20 (27)	143 (100)	< 0.001
**Laboratory findings**				
Lactic acid (mmol/L, n = 220)	2.86 (1.90–4.20)	1.67 (1.26–1.91)	3.58 (2.66–5.17)	< 0.001
CRP (mg/dL, n = 215)	13.07 (5.80–24.17)	13.58 (5.35–24.67)	12.93 (5.84–24.17)	0.994
PCT (ng/mL, n = 181)	5.24 (0.87–21.67)	1.04 (0.27–5.50)	8.07 (1.50–34.17)	< 0.001
Exosomal CD63 (µg/mL)	78 (35–130)	42 (22–90)	90 (49–140)	< 0.001
**Severity of illness**				
SAPS 3 score	54 (47–62)	49 (40–57)	57 (51–65)	< 0.001
APACHE II score	24 (19–29)	23 (17–28)	24 (20–30)	0.028
SOFA score	9 (6–11)	6 (4–8)	10 (8–12)	< 0.001
**Mortality**				
28-day ICU mortality	37 (18)	12 (16)	25 (18)	0.712
In-hospital mortality	51 (24)	17 (23)	34 (24)	0.999
90-day ICU mortality	70 (32)	22 (30)	48 (34)	0.675

Data are presented as medians (interquartile range) or numbers (%).

BMI, body mass index; ICU, intensive care unit; CRP, C-reactive protein; PCT, procalcitonin; SAPS, simplified acute physiology score; APACHE, acute physiology and chronic health evaluation; SOFA, sequential organ failure assessment.

**Figure 1:**
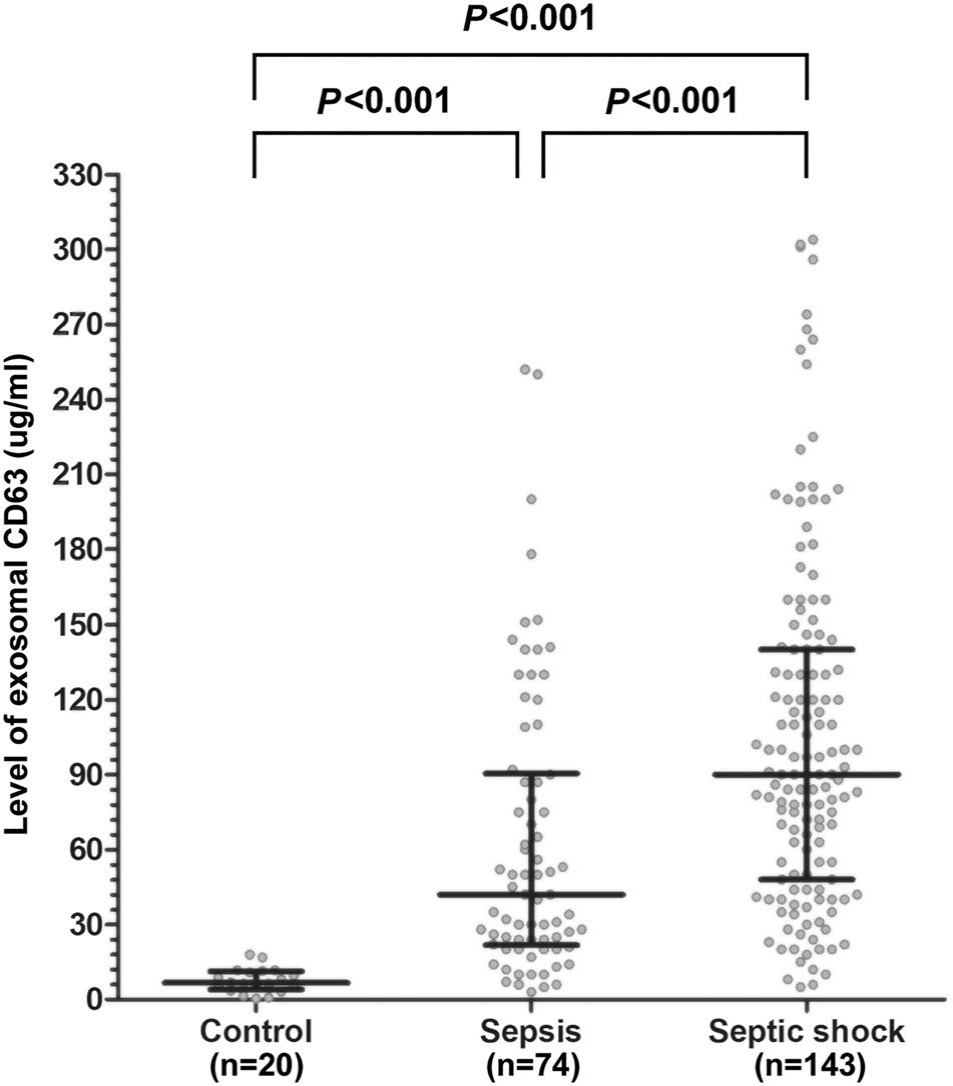
Exosomal CD63 levels in control, sepsis, and septic-shock groups. The line in the middle indicates the median and lines in the top and bottom indicate the interquartile ranges of exosomal CD63 levels.

The exosomal CD63 was measured in all participants. The median exosomal CD63 levels were 78 µg/mL (35–130 µg/mL). The level of exosomal CD63 was higher among patients with septic shock (90 µg/mL, IQR 49–140 µg/mL) compared with healthy controls (6.6 µg/mL, IQR 4.8–11 µg/mL) and sepsis patients without shock (42 µg/mL, IQR 22–90 µg/mL, p < 0.001; Fig. 1).

**Figure 2:**
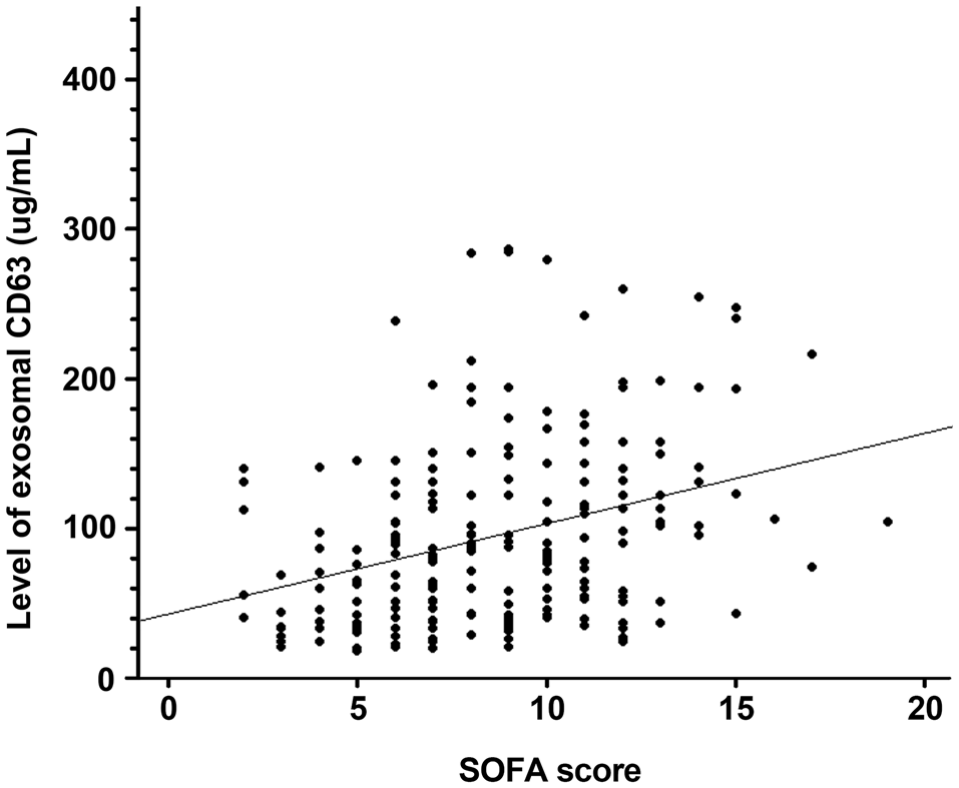
Correlation between exosomal CD63 levels and sequential organ failure assessment (SOFA) scores in patients with sepsis. Slope: 6.73 (95% confidence interval (CI); 4.28–9.19), r^2^: 0.12, Pearson’s r: 0.35 (95% CI; 0.22–0.46, p < 0.001).

**Figure 3:**
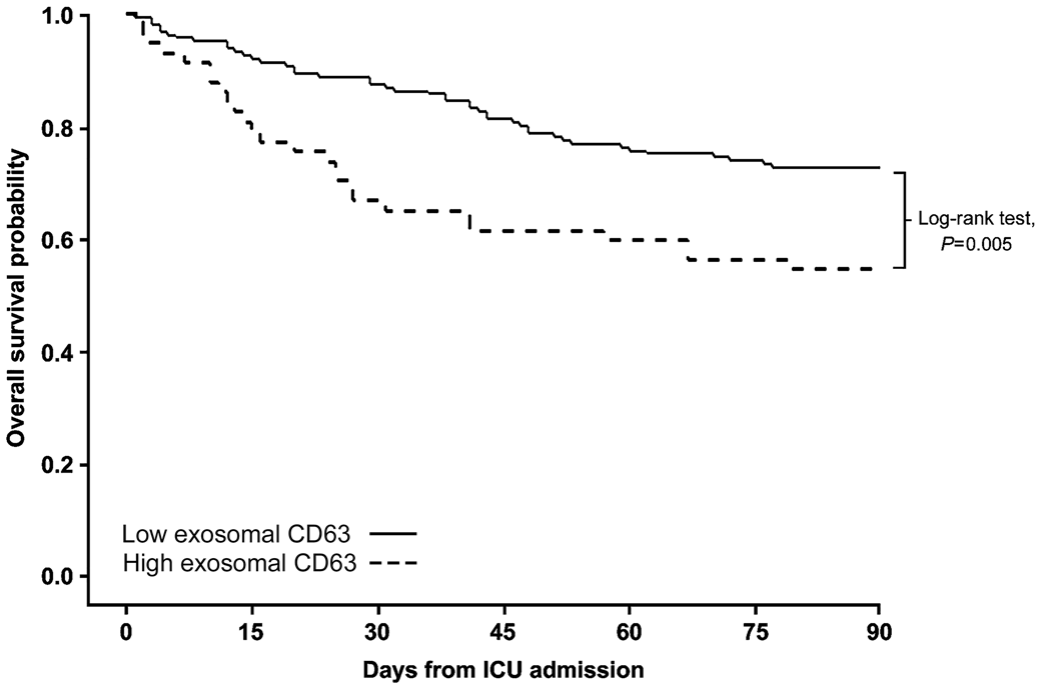
Kaplan–Meier survival estimation of patients with high and low-exosomal CD63 levels (Log-rank p = 0.005).

Linear regression method was used to assess the association between exosomal CD63 levels and severity of organ failure. A positive correlation between exosomal CD63 and SOFA scores was observed in patients with sepsis (*r* value = 0.35; 95% confidence interval (CI) 0.22–0.46) (Fig. 2). We categorized patients as two groups of CD63 levels > than 126 µg/mL and < 126 µg/mL, and correspondingly, compared the initial diagnosis, clinical status, severity of illness, and mortality between the two groups. The group with higher exosomal CD63 levels was significantly associated with septic shock, requirement for mechanical ventilation or vasopressor support, and the severity of illness calculated using SAPS 3, APACHE II, and SOFA scores, 28-day, in-hospital, and 90-day mortalities (Table 2). Moreover, the Kaplan–Meier survival method showed a significant difference in 90-day survival between patients with high- and low-exosomal CD63 levels (log-rank p = 0.005) (Fig. 3).

**Table 2:** Outcomes and illness severity among patients dichotomized by exosomal CD63 level ≥ 126 and < 126 µg/mL.

Label	Low CD63 (n = 160)	High CD63 (n = 57)	p*-*value
**Diagnosis**			
Sepsis	61 (38)	13 (23)	0.036
Septic shock	99 (62)	44 (77)	
**Clinical status on ICU admission**			
Need for mechanical ventilation	57 (36)	31 (54)	0.020
Need for vasopressor support	113 (71)	50 (88)	0.017
**Severity of illness**			
SAPS 3 score	52 (45–60)	57 (53–68)	0.002
APACHE II score	24 (19–28)	26 (19–33)	0.040
Initial SOFA score	8 (6–10)	10 (8–12)	< 0.001
**Mortality**			
28-day mortality	18 (11)	19 (33)	< 0.001
In-hospital mortality	30 (19)	21 (38)	0.010
90-day mortality	44 (28)	26 (46)	0.019

Data are presented as median (interquartile range) or number (%).

ICU, intensive care unit; SAPS, simplified acute physiology score; APACHE, acute physiology and chronic health evaluation; SOFA, sequential organ failure assessment.

## Discussion

To our knowledge, this is the first research to comprehensively assess the association between exosomal CD63 levels and severity of organ dysfunction and mortality in critically ill patients with sepsis. The result of this study indicated that exosomal CD63 levels were associated with the severity of organ dysfunction and prediction of death.

Tetraspanins have been known to participate in a wide spectrum of physiological and pathological processes of the immune response to infections^12,14,27^. Among them, CD63 interacted with many different pathogens, cells, and proteins, either directly or indirectly, as this is not only highly expressed in the transmembrane domain of exosomes but is also located in intracellular organelles^28,29^. Growing evidence suggested that exosomal CD63 participated in intracellular transport of diverse pathogens to hosts^16–20^. Beatty et al. demonstrated a trafficking pathway from CD63-positive exosomes to the bacterial inclusion as exosomal CD63 was localized in *Chlamydia-trachomatis*-infected cells by confocal microscopy analysis^16^. In the same manner, reduction in adherence of *Neisseria meningitides* to human epithelial cells was also noted in pretreated epithelial cells with an anti-CD63 antibody or small interfering RNA^17^. Moreover, CD63 has been extensively studied as a general mediator of virus infection, including the human papillomavirus, human immunodeficiency virus-1, and hepatitis C virus. These studies have demonstrated that exhaustion of CD63 or anti-CD63 antibodies treatment could reduce the infectivity of the virus^18–20^. These findings suggested that exosomal CD63 may participate in triggering infection and may influence the host’s immune system.

As discussed in previous studies, exosomal CD63 was also involved in multiple processes of inflammatory responses to infection. Neutrophil-derived exosomal CD63 increased the retention of precursor of neutrophil elastase in exosomes, a component of neutrophil extracellular traps^30–32^. Formation of neutrophil extracellular traps were vital to pathogen clearance, but simultaneous neutrophil extracellular traps induced collateral damage to host tissues and also correlated with the severity and mortality in sepsis^31,33,34^. Additionally, CD63 participated in the adhesion, morphological changes, and spreading of monocytic cells to induce various types of multinucleated giant cells to serve in the front-line of host defense, and to trigger an immune-inflammatory response to infection^35–37^. Similarly, the expression of CD63 was related with platelet activation and its interaction with leukocyte and endothelial cells that promoted platelet consumption and coagulopathy, and overproduced proinflammatory cytokines, finally leading to apoptosis of cell and multiorgan failure^38–41^. Taken together, these immune cells activated by CD63 may cause inflammation-induced organ damage, thus leading to multiorgan failure and eventually to death. On this subject, we aimed to analyze the relationship between exosomal CD63 and severity and mortality of sepsis in humans.

The strength of our study is attributed to the capacity to quantify exosomal CD63 in a larger cohort of critically ill patients with sepsis. Compared with other studies, the overall level of exosomal CD63 in sepsis patients was associated with organ failure and mortality in this study, thus suggesting its possibility as a biomarker for the assessment of the severity and for the prediction of mortality owing to sepsis. Nevertheless, additional multicenter studies are needed to validate its efficacy and reliability as a biomarker for sepsis. In addition, considering the fact that tetraspanins contribute to multiple pathological processes that might be therapeutically targeted^42^, it seems that it is necessary to analyze a) the competency of CD63 as a single biomarker compared with the known markers or scoring systems, and b) the therapeutic role of CD63 for sepsis based on the use of anti-CD63 antibodies, tetraspanin-derived recombinant soluble extracellular loops, and RNA interference knockdown strategies. Furthermore, there are several limitations associated with our study. First, the study was conducted at a single referral center that may limit the generalizability of the data. Second, other exosomal tetraspanins were not analyzed as potential biomarkers in this study. Previous studies have illustrated that various tetraspanins influenced the pathogenesis of the immune and host responses in diverse ways^12–14,27^. Although quantification of exosomal CD63 may be an advantage and the point of differentiation in our study, additional studies, including other tetraspanins, could strengthen the analysis and diagnostic potential of tetraspanins in sepsis. Combining tetraspanins with other biomarkers may provide more insights in the understanding of the immune/inflammatory interplay that is ill-defined in sepsis, and will aid in the interpretation of their role as diagnostic and prognostic biomarkers in sepsis. Finally, we tried to explain that CD63 is involved in immune and inflammation responses to infection and associated with sepsis severity and mortality through the results of previous studies. However, sepsis/septic shock could also directly increase CD63; the mechanism of increased CD63 levels in the exosomes of critically ill patients is still unclear. Further studies, including biogenetic mechanisms and functional studies of exosomal CD63 would provide helpful clues for the development of new targeted treatment based on exosomal CD63 modulation in sepsis.

In summary, exosomal CD63 levels were associated with the severity of organ failure and predictive mortality in critically ill patients with sepsis.

## Data Availability

The data that support the findings of this study are available on request from the corresponding author. The data are not publicly available due to privacy or ethical restrictions.

## Abbreviations

APACHE II: Acute Physiology and Chronic Health Evaluation II
ICU: Intensive care unit
SAPS 3: Simplified acute physiology score 3
SOFA: Sequential Organ Failure Assessment
